# The role of oxidized lipid species in insulin resistance and NASH in children

**DOI:** 10.3389/fendo.2022.1019204

**Published:** 2022-10-03

**Authors:** Nicola Santoro, Ariel E. Feldstein

**Affiliations:** ^1^ Department of Pediatrics, Kansas Medical Center, Kansas City, KS, United States; ^2^ Department of Medicine and Health Sciences, “V.Tiberio” University of Molise, Campobasso, Italy; ^3^ Department of Pediatrics, Yale University School of Medicine, New Haven, CT, United States; ^4^ Department of Pediatrics, University of California San Diego, La Jolla, CA, United States; ^5^ Global Drug Discovery, Novo Nordisk, Copenhagen, Denmark

**Keywords:** lipids, PUFA (polyunsaturated fatty acids), NAFLD (nonalcoholic fatty liver disease), diabetes, children

## Abstract

During the last two decades, nonalcoholic fatty liver disease (NAFLD) has emerged as the most common hepatic disease in pediatrics, mainly owing to the rising prevalence of pediatric obesity. Epidemiological studies have shown that the progressive increase in NAFLD prevalence is associated not only with obesity but also with changes in dietary habits experienced by all age groups, characterized by the increased intake of added sugars and certain fatty acids. In this review article, we focus on the effect of oxidized fatty acids deriving from linoleic acid and arachidonic acid on the pathogenesis and progression of NAFLD in youth.

## Clinical and histopathological features of pediatric NAFLD

The term nonalcoholic fatty liver disease (NAFLD) is used to define a syndrome affecting the liver and spanning from the accumulation of triglycerides in the hepatocytes (steatosis) to cirrhosis and hepatocellular carcinoma with steatohepatitis (NASH) and fibrosis representing intermediate stages (1–5). From a pathophysiologic point of view, insulin resistance (IR) plays a key role in the onset of the process and probably also in its progression (1, 6, 7), but the temporal relationship between these two phenomena is difficult to untangle. It has been shown, in fact, that youth with NAFLD tend to show a greater degree of IR than youth with similar degrees of adiposity, visceral fat, and intramyocellular lipids (8). These data demonstrate the effect of NAFLD on IR, but longitudinal studies also show that youth with obesity who develop NAFLD over time tend to have higher degrees of IR as compared with those who do not develop NAFLD (9, 10). Therefore, the relationship between these two entities is rather complex, but it is likely that IR precedes the onset of intrahepatic fat accumulation and the latter eventually contributes to perpetuating IR (9). This is supported by the fact that youth with NAFLD tend to show a higher prevalence of conditions related to IR, such as high triglycerides (TGs) and LDL, and a high prevalence of prediabetes and type 2 diabetes (T2D) (9, 11–13). This phenotype closely resembles that of metabolic syndrome; therefore, some authors have proposed the term metabolic associated fatty liver disease (14) to indicate the metabolic component of the disease (15, 16). The key issue is that early onset NAFLD seems to have a faster and more aggressive progression than the disease in adults. Youth who develop NAFLD around age 13 have, in fact, have 13.6 times higher risk than disease-free youth of similar age and gender to develop end-stage liver disease in their early 20s (17). Moreover, recently, Simon et al. showed in an ~16-year follow-up study that early onset NAFLD is associated with higher rates of cancer-, liver-, and cardiometabolic-specific mortality compared with matched general population controls (14). These data are in agreement with earlier data in youth showing that pediatric NAFLD is associated with an adverse cardiovascular profile characterized by an increase in small-dense lipoprotein particles and a decrease in HDL-cholesterol (18).

This may be because inflammation and fibrosis develop quite early in youth with NAFLD (17). NASH is a key component of the disease as it drives its progression; NASH is triggered by free fatty acids (FFA) that are not esterified into TGs in the liver (19). These FFA bind the TLR-4 receptor on the membrane of liver macrophages starting a cascade of events that leads to tissue damage and fibrosis (19, 20). Another important factor to take into account is that, from a histological point of view, pediatric NAFLD seems to be slightly different from the adult type as it is characterized by chronic portal inflammation (that is absent or mild in adult NASH) and early onset fibrosis (21). In fact, inflammation in adults is localized mainly in the centrilobular portion of the hepatic lobule (borderline zone 3), whereas in youth, the inflammation is localized in the periportal portion of the hepatic lobule (borderline zone 1) (21). How and whether this different distribution contributes to the progression of the disease in youth remains unknown. A reason for this difference may be the different compartmentalization of the pediatric liver as compared with the adult liver, but there are no data supporting this hypothesis.

Steatohepatitis further worsens the degree of insulin resistance, and as the inflammation worsens, the prevalence of prediabetes and T2D increases (11). Importantly, the prevalence of NAFLD differs among different races and ethnicities, with Hispanic people showing the highest rates and non-Hispanic Black (NHB) people showing the lowest rates (9). The latter group seems to be protected against intrahepatic accumulation despite the degree of obesity and IR (15). However, when NHB develop NAFLD (about 13% of NHB with obesity), they show a more severe degree of IR and higher rates of prediabetes and T2D than Hispanic people (16).

## Omega-6 polyunsaturated fatty acid (PUFA)-derived oxylipins

Over the last centuries, the amount and quality of fatty acids introduced into the diet have changed (22). In particular, there has been a progressive increase in omega-6 polyunsaturated fatty acids (n-6 PUFA) (22). The main n-6 PUFA is linoleic acid (LA), a fatty acid that can be introduced only through the diet and, therefore, is indicated as an “essential” fatty acid. This is indispensable for brain development and cell membrane formation; therefore, human milk is rich in LA (23). There should be a balance between n-6 and n-3 PUFA, and the ratio between n-6 and n-3 PUFA should be 1:1 (22). This would be the ideal, but it is in practice unrealistic, and the American Heart Association recommends this ratio to be 4:1. Current nutritional habits, though, are far from what is recommended, and the n-6/n-3 PUFA ratio in the American diet is much higher (about 15/1) (24, 25). This is important because n-3 PUFA carries more beneficial effects and are able to counterbalance the detrimental consequences of an excess of n-6 PUFA (26). The main n-3 PUFA is alpha-linoleic acid (ALA), and it is also an essential fatty acid. The n-6 and n-3 PUFA go through the same enzymatic pathway, but the products obtained are different and have different metabolic effects (27). The main product of LA is arachidonic acid (AA), which is a key precursor of prostaglandins, thromboxanes, and leukotrienes (27). Importantly, under certain conditions, such as the subtle inflammation present in individuals with obesity, LA and AA are oxidized through enzymatic and non-enzymatic mechanisms into oxylipins, such as octadecadenoic and oxo-octadecadenoic acids, derived from LA (OXLAM), and hydroxy-eicosatetraenoic acids derived from the oxidation of AA (OXAA).

Studies in youth and adults with NAFLD show that oxylipins plasma concentrations are associated with liver inflammation and injury in individuals with NAFLD/NASH. Studies in adults also show that individuals with biopsy-proven NASH tend to have higher circulating OXLAM than individuals without NASH (28). A similar effect is shown in youth with obesity and NAFLD (29). In fact, in youth with NAFLD and OXLAM are associated with the plasma concentrations of CK-18 and ALT, two biomarkers of liver injury, whereas this association is not present in individuals without NAFLD (29). These data suggest that oxylipins may contribute to or lead to inflammation when NAFLD occurs. This is possible because of the great amounts of n-6 PUFA in the Western diet tend to accumulate in the liver. To date, though, it is still unclear whether LA and AA are accumulated in the liver and then converted into OXLAM and OXAA or if the conversion happens in the adipose tissue and then the oxidized FFA reache the liver. Although this was never proven, the latter hypothesis is not unlikely given the presence of the so-called “sterile inflammation” (or subtle inflammation) in the adipose tissue of individuals with obesity (30). On the other hand, studies looking at the histology of the liver in individuals with NAFLD show that the liver of individuals with NAFLD/NASH is characterized by high concentrations of intrahepatic n-6 PUFA and a low amount of n-3 PUFA (31). Nutritional studies also show that the intake of AA is associated with liver fibrosis in Hispanic youths with NAFLD, and this evidence further corroborates the link between high n-6 PUFA intake and liver injury in the context of NAFLD (32).

Genetic background also modulates the association between oxylipins and NAFLD. In fact, the strongest genetic determinant of NAFLD, the rs738409 variant (33), affects the association between oxylipins and NAFLD with individuals homozygous for the minor at-risk allele (G) showing a strong association between markers of liver injury and intrahepatic fat content (29). This association is weaker or absent in the other genotypes (26). On the other hand, it is of note that oxylipin concentrations are also dependent on the *FADS* haplotype (34). *FADS* is the gene coding for the fatty acid desaturase, a rate-limiting enzyme in the processing of the n-6 PUFA. A study in youth shows that the haplotype associated with lower enzymatic activity (AA) determines high concentrations of LA and OXLAM and lower conversion of LA into AA in youth with obesity (34).

**Figure 1:**
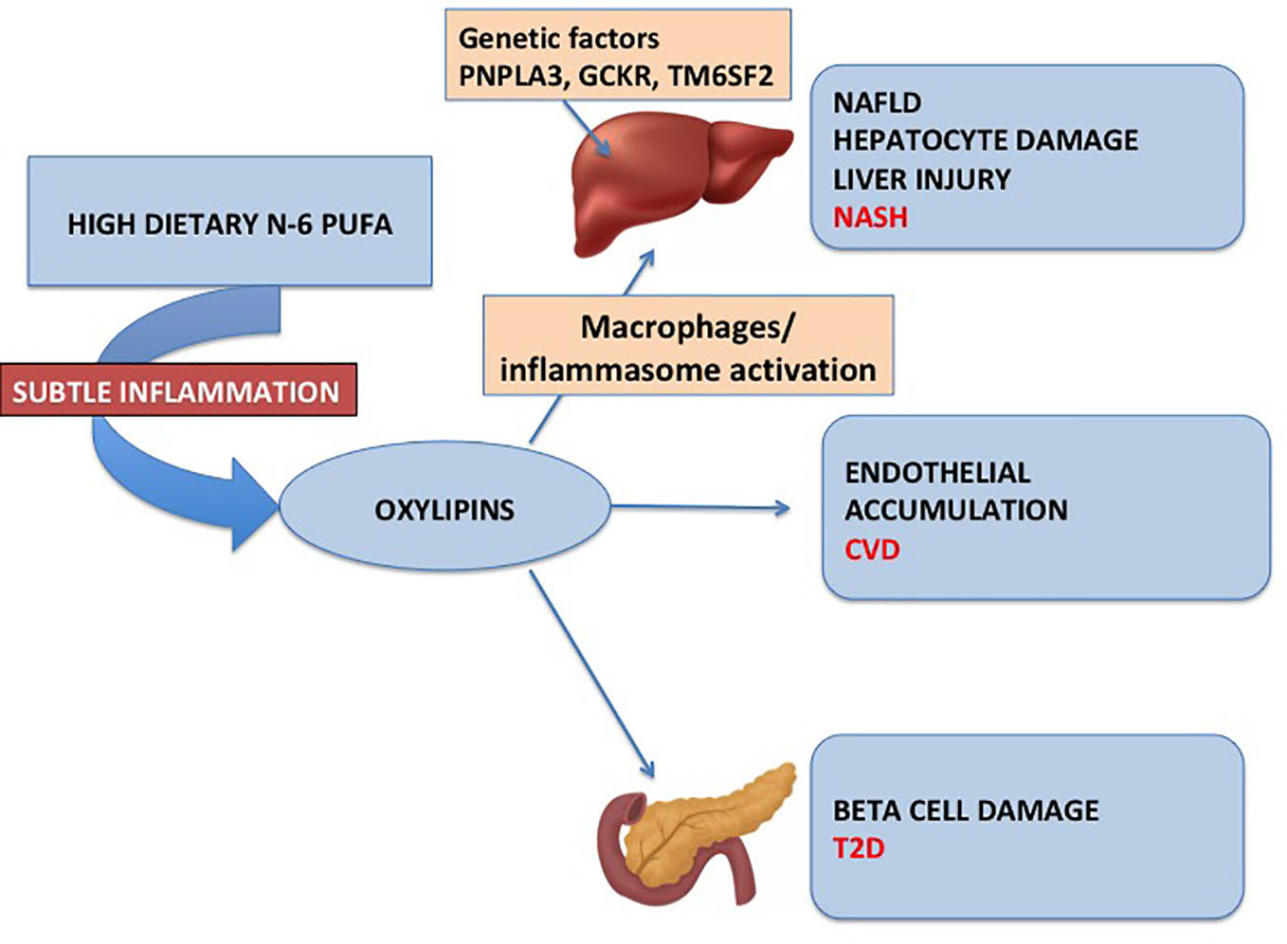
The figure depicts the mechanism through which excess n-6 PUFA and lack of n-3 PUFA in the diet may predispose to NAFLD, diabetes, and cardiovascular disease.

Importantly, it is shown that the detrimental effect of oxylipins in youth with obesity may not be limited to the liver but may extend to the pancreatic beta cell (29). In fact, plasma oxylipin concentrations are associated with a lower disposition index, a biomarker of insulin secretion adjusted by insulin sensitivity, and that youth with T2D have higher plasma concentrations of oxylipins (26). This observation may suggest that oxylipins may be a pathogenic link between NAFLD and diabetes (**Figure 1**).

More recently, some studies have pointed out that maternal PUFA plasma concentrations affect the liver metabolism of infants. In fact, Wahab et al. show that maternal low n-3 PUFA and high n-6 PUFA plasma concentrations during pregnancy are associated with the accumulation of fat in the liver during early childhood in the offspring (35).

## Mechanisms linking oxylipins to liver injury: The role of inflammasomes

Although oxidative stress is recognized as a key mechanism contributing to hepatocyte injury during NASH development, the direct mechanisms by which oxylipins contribute to liver injury in this context remain incompletely understood. In a recent study (36), we used three different isocaloric high-fat diets containing different amounts of LA (low and high) or enriched with oxylipins to mechanistically understand their impact on the development of liver injury. We further aimed to test whether oxylipins directly modulate the oxidative stress response and innate immunity. The findings identified a role of oxylipins in the activation of the NLRP3 inflammasome linking lipid metabolism with innate immune responses, cell death, and inflammation.

## Reversing oxylipins ameliorates NAFLD phenotype and insulin resistance

Given the association between oxylipins and NAFLD/NASH, some studies have targeted these compounds to try to ameliorate NAFLD/NASH. In a clinical trial, Zein et al. show that pentoxifylline is effective in reducing the plasma concentrations of OXLAM and OXAA (37). In particular, the authors enrolled 47 subjects in a 12-month double-blind placebo-controlled clinical trial (37). Of them, 21 were given pentoxifylline, and 26 were given placebo (37). Individuals taking pentoxifylline showed a marked reduction of OXLAM and OXAA, although changes in their progenitors, the LA and AA, respectively, were not different between the groups (37). More interestingly, changes in OXLAM and OXAA were associated with an improvement in liver fibrosis and inflammation evaluated through liver biopsy (37). Despite the interest generated by these data, to date, it is still unclear how pentoxifylline acts. One theory is that it could reduce oxidation occurring during inflammation, reducing the generation of oxygen-derived free radicals and OXLAM and OXAA (37).

It has also been observed that pentoxifylline has an inhibitory effect on hepatic macrophage M1 polarization in high fat diet–induced NAFLD, thus suggesting a potential molecular mechanism by which it could ameliorate fatty liver disease (38).

More recently, a proof-of-concept dietary intervention aimed at reducing the ratio between n-6 and n-3 PUFA in the diet has been published (39). Seventeen youth with obesity and NAFLD underwent a low n-6/n-3 PUFA diet for 12 weeks (39). The study showed that an n-6/n-3 PUFA ratio in the diet of 1 to 4 for 12 weeks is associated with the decline in OXLAM (measured in plasma every 4 weeks) and an ~32% reduction of intrahepatic fat content (39). Interestingly, this study showed also an improvement in insulin sensitivity and glucose tolerance. In fact, three out of the four youth with prediabetes reverted their clinical condition at the end of the intervention (39). A follow-up study has also shown that lowering the n-6/n-3 PUFA ratio causes the amelioration of insulin clearance independent of changes in the hepatic content (40). This further strengthens the assumption that changes in OXLAMs can directly affect insulin metabolism. Despite this evidence, also for the dietary intervention, it is unclear what could be the mechanism leading to these changes. It can be hypothesized that lowering the intake of the substrate (LA) may reduce the formation of OXLAM and also that, when the n-6 PUFA concentration is not overwhelmingly higher than the n-3 PUFA concentration, the latter may better counterbalance the proinflammatory effect of n-6 PUFA.

## Conclusions

The data present in the literature about the role of n-6 PUFA in the pathogenesis of NAFLD and T2D clearly suggest that therapeutic efforts should be conducted to reduce plasmatic n-6 PUFA and possibly to reduce n-6 PUFA intake in order to prevent those diseases.

## Author contributions

All authors listed have made a substantial, direct, and intellectual contribution to the work, and approved it for publication.

## Funding

AF is funded by the National Institutes of Health grants R01 DK113592 and R01AA028134. NS is funded by the National Institutes of Health grants R01 DK111038 and R01 MD015974.

## Conflict of interest

AF is an employee and stockholder of Novo Nordisk.

The remaining author declares that the research was conducted in the absence of any commercial or financial relationships that could be construed as a potential conflict of interest.

## Publisher’s note

All claims expressed in this article are solely those of the authors and do not necessarily represent those of their affiliated organizations, or those of the publisher, the editors and the reviewers. Any product that may be evaluated in this article, or claim that may be made by its manufacturer, is not guaranteed or endorsed by the publisher.
