# Corrigendum: The role of oxidized lipid species in insulin resistance and NASH in children

**DOI:** 10.3389/fendo.2024.1461564

**Published:** 2024-12-19

**Authors:** Nicola Santoro, Ariel E. Feldstein

**Affiliations:** ^1^ Department of Pediatrics, Kansas Medical Center, Kansas City, KS, United States; ^2^ Department of Medicine and Health Sciences, “V.Tiberio” University of Molise, Campobasso, Italy; ^3^ Department of Pediatrics, Yale University School of Medicine, New Haven, CT, United States; ^4^ Department of Pediatrics, University of California San Diego, La Jolla, CA, United States; ^5^ Global Drug Discovery, Novo Nordisk, Copenhagen, Denmark

**Keywords:** lipids, PUFA (polyunsaturated fatty acids), NAFLD (nonalcoholic fatty liver disease), diabetes, children

In the published article, there was an error regarding the affiliations for Ariel E. Feldstein. As well as having affiliation 4, they should also have “Global Drug Discovery, Novo Nordisk, Copenhagen, Denmark”.

In the published article, there was an error in the **Conflicts of interest**, and a declaration regarding author Ariel E. Feldstein was erroneously excluded.

The corrected **Conflicts of interest** section appears below.

“AF is an employee and stockholder of Novo Nordisk.

The remaining author declares that the research was conducted in the absence of any commercial or financial relationships that could be construed as a potential conflict of interest.”

The author apologizes for this error and states that this does not change the scientific conclusions of the article in any way. The original article has been updated.

